# Knowledge, attitude, and practice of menstrual blood-derived mesenchymal stem cells among female healthcare workers in India

**DOI:** 10.3389/fpubh.2023.1102016

**Published:** 2023-05-04

**Authors:** Karuna Nidhi Kaur, Dhruva Nandi, Krithika Ramachandran, Lita Mohanan, S. Subhashini, Mehak Segan, Saswaty Tripathy, Rajiv Janardhanan

**Affiliations:** ^1^Amity Institute of Public Health, Amity University, Noida, India; ^2^SRM Medical College Hospital and Research Centre, SRM University, Kattankulathur, India; ^3^Apollo Hospital, Chennai, India

**Keywords:** public health, women health, stem cell, health care profession, mesenchymal stem cells, India

## Abstract

**Introduction:**

Mesenchymal stem cells (MSCs) are pluripotent progenitor cells that can be differentiated into a variety of specialized cell types. Menstrual blood, such as umbilical cord blood and bone marrow stem cells, is a rich source of MSCs with proliferative properties. This research was conducted to understand the knowledge, attitude, and practice of menstrual blood donation regarding menstrual blood-derived mesenchymal stem cells (MenSCs) among female healthcare workers in India.

**Methodology:**

A cross-sectional online and offline survey was conducted at the national level between 20 November 2021 and 10 March 2022. A self-constructed semi-structured questionnaire was distributed through Google Forms on various social media platforms. The questionnaire was self-administered, and data were collected using the purposive sampling technique.

**Results:**

A total of 499 respondents completed the questionnaire. Nearly 49% of the respondents had adequate knowledge, 54% showed a positive attitude, and 45% reported adequate practices regarding menstrual blood donation and the usage of related products. The educational background, occupational status, and monthly income of the participants were found to be significantly associated with their attitude toward MenSCs.

**Conclusion:**

There is a need to promote interactive sessions on MenSCs among healthcare professionals in order to bridge the gap between general populations and the healthcare setting. Enhancing knowledge and awareness regarding the potential benefits of MenSCs would help in dissipating the age-old myths associated with menstruation and will benefit society as a whole.

## Introduction

Mesenchymal stem cells (MSCs) are pluripotent progenitor cells capable of differentiating into a variety of specialized cell types such as osteoblasts, chondrocytes, and adipocytes (1, 2). MSCs can be extracted from a range of adult tissues including, but not limited to, the bone marrow, peripheral blood, adipose tissue, umbilical cord, placenta, and menstrual blood (3–5). Over the last 12 years, MSCs have gained attention from researchers worldwide owing to their high proliferative rate, low immunogenicity, non-invasive collection techniques, high abundance, unlimited availability, and fewer ethical issues (6, 7). MSCs are a novel and easily accessible therapeutic tool for regenerative medicine and tissue engineering. The use of MSCs for the treatment of different ailments is progressing at a rapid pace, and their potential applications are expanding.

Menstrual blood is an expandable source of such stem cells with a high proliferative capacity and restorative properties compared to umbilical cord blood and bone marrow stem cells. Researchers have successfully retrieved stem cells from menstrual blood, and this makes the concept of stem cell preservation viable for all women, even those who never give birth (8).

The identification of stem cells in the menstrual blood will give women a new perspective on menstruation, which has been stigmatized in low-and middle-income countries (LMICs) such as India (9–11). This type of novel revelation can be a boon for the Indian scenario, where approximately 355 million menstruating girls and women reside. Unfortunately, the issue of menstruation has been a taboo for centuries in India, with menstruating women deemed filthy and forced to live under harsh limitations, prohibited from social and religious gatherings, temples, and shrines (9).

Therefore, it is imperative to raise awareness and knowledge among women from all strata regarding menstrual blood-derived mesenchymal stem cells (MenSCs). Although menstrual blood is a cost-effective, convenient, and easily accessible source of stem cells, minimal research has been conducted to assess the knowledge gap as well as the perception of women toward MenSCs. This becomes particularly important among healthcare workers as they are the link connecting healthcare centers and community resources. Hence, this research was conducted to understand the knowledge, attitude, and practice of donating menstrual blood among female healthcare workers in India.

## Methodology

### Participants and recruitment

A cross-sectional online and offline survey was conducted at the national level between 20 November 2021 and 10 March 2022. The purposeful sampling technique was used for data collection. The questionnaire was distributed through Google Forms and physical copies to a total of 550 participants for filling the questionnaire after reading a descriptive introduction on the topic. The link to the Google Forms was shared on various social media platforms. Women above the age of 18 years who were in the healthcare profession being a student or a worker were included. Healthcare workers who had menopause and who identified themselves as males were excluded from the study. No incentive was provided for questionnaire completion. This research was approved by the SRM Medical and Research Ethics Clearance Subcommittee. After taking informed consent, women aged 18 years and above were recruited from various colleges and healthcare setups to complete the questionnaire.

### Survey

A self-constructed semi-structured questionnaire was made based on an extensive review of relevant literature (12, 13). A pilot study was conducted among 15 participants, who volunteered and gave informed consent after going through the study objectives, to gain inputs from the participants to improve the clarity and ensure the validity of the questionnaire. However, the participants enrolled in the pilot study were not part of the survey, and their responses were not incorporated in the results of this manuscript. The questionnaire was further validated by a gynecologist and then used in the survey. The questionnaire covered questions related to socio-demographic characteristics and the assessment of knowledge, attitude, and practice regarding MenSCs. The survey was anonymous, and ethical clearance was obtained from the institutional ethical clearance committee. This questionnaire was copyrighted by the authors with the diary number 32063/2021-CO/L.

### Data analysis

Data were entered in an Excel sheet, and statistical analysis was performed using SPSS software version 25. Descriptive statistics, such as frequency, were used to present the socio-demographic characteristics of the respondents and various factors included in the assessment of knowledge, attitude, and practice regarding MenSCs. Pearson’s chi-square test was performed to find the significant associations between socio-demographic characteristics and the factors included in this study.

## Results

Out of 550 participants, a total of 499 participants completed the questionnaire. In total, 67% of the participants were from 18 to 24 years (Table 1). Out of the 499 female participants, 78% of the respondents were either pursuing or had completed their bachelor’s degree; 63% of the respondents were from the medical or allied health sciences department, while 37% had a paramedical background. The majority of the respondents were students (57.5%), and 35% had private-sector jobs. A total of 53% of the respondents earned 10 to 29,000 rupees per month, whereas only 16% earned 50,000–1,00,000 rupees per month. The proportion of unmarried respondents in our study was 76%, and only 22% were reported to be married. In total, 69% of the participants were practicing Hinduism, and a majority of the respondents were from the southern region of India (52.1%), followed by northern India (39.2%).

**Table 1:** Socio-demographic characteristics of the respondents (*n* = 499).

Characteristics	Sub-group	Number (%)
Age (years)	18–24	334 (66.9)
	25–39	155 (31.1)
	40 and above	10 (2.0)
Gender	Female	499 (100)
Education	Graduate^*^	389 (78.0)
	Post-graduate^**^	95 (19.0)
	Doctorate (PhD)	15 (3.0)
Department	Medical/Allied Health Sciences	313 (62.7)
	Paramedical	186 (37.3)
Occupation	Employed	204 (40.9)
	Unemployed	295 (59.1)
Profession	Student	287 (57.5)
	Private sector	174 (34.9)
	Government sector	21(4.2)
	Self employed	17 (3.4)
Family monthly income (Indian rupees) (14)	10–29 thousand	263 (52.7)
	30–49 thousand	154 (30.9)
	50 thousand – 1 lakh	79 (15.8)
	1 lakh and above	3 (0.6)
Marital status	Unmarried	381 (76.4)
	Married	108 (21.6)
	Divorced	6 (1.2)
	Separated	2 (0.4)
	Widowed	2 (0.4)
Religion	Hindu	345 (69.1)
	Muslim	76 (15.2)
	Christian	57 (11.4)
	Sikh	13 (2.6)
	Others^***^	8 (1.6)
Native region	North region	196 (39.2)
	East region	31 (10.2)
	West region	9 (1.8)
	South region	260 (52.1)
	Central region	1 (0.2)

* Pursuing or completed the bachelor’s degree.

** Pursuing or completed the master’s degree.

*** Jain, Buddhist, Parsi, Atheist, etc.

**Figure 1:**
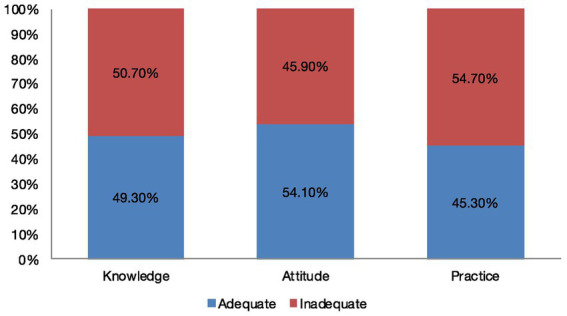
Knowledge, attitude, and practice level among respondents regarding menstrual blood-derived stem cells. This figure depicts the level of knowledge, attitude, and practice among 499 respondents toward MenSCs. In total, 49% of the respondents had an adequate knowledge level, 54% showed a positive attitude, and 45% reported adequate practices regarding menstrual blood donation and the usage of related products.

A total of 49% of the respondents in our study had adequate knowledge regarding MenSCs (Figure 1). However, 53% of the respondents were unaware that menstrual blood can be donated (Table 2). Educational courses were the predominant source of obtaining knowledge related to MenSCs (50.6%). Only 31% of the respondents were familiar with the notion that MenSCs can be utilized for various therapeutic purposes. Similarly, 43% of the respondents were unaware of the novel regenerative properties of the MenSCs, and 24% were unaware of mesenchymal stem cell banking in India. In addition, 83% of the respondents showed an interest toward enhancing their knowledge with respect to MenSCs.

**Table 2:** Knowledge of the respondents regarding menstrual blood-derived stem cells.

Questions	Options	Number (%)
Do you know that menstrual blood can be donated?	Yes	141 (28.3)
	No	266 (53.3)
	Do not know	92 (28.3)
Do you know about menstrual blood-derived stem cells?	Yes	168 (33.7)
	No	243 (48.7)
	Do not know	88 (17.6)
If yes, how do you know about menstrual blood-derived stem cells?	Colleague	27 (16.0)
	Educational courses	85 (50.6)
	Social media^*^	49 (29.2)
	Non-respondents	7 (4.2)
Do you know that stem cells from menstrual blood can be used to treat many diseases?	Yes	152 (30.5)
	No	224 (44.9)
	Do not know	123 (24.6)
Do you know that stem cells derived from menstrual blood can be used for regenerative therapy?	Yes	156 (31.3)
	No	213 (42.7)
	Do not know	130 (26.1)
Are you aware about mesenchymal stem cell banking in India?	Yes	122 (24.4)
	No	234 (46.9)
	Do not know	143 (28.7)
Would you be interested in enhancing your knowledge about menstrual blood-derived stem cells?	Yes	414 (83.0)
	No	53 (10.6)
	Do not know	32 (6.4)

* YouTube, Instagram, Twitter, WhatsApp, and Facebook.

In total, 54% of the respondents in our study were reported to have a positive attitude toward menstrual blood donation and the utilization of MenSCs for therapeutic purposes (Figure 1). Impressively, 65% of the respondents agreed that menstrual blood donation can be a boon to society, 73% supported the fact that menstrual blood for MSC collection should be promoted, and 82% felt that government should take proactive actions toward increasing MenSCs awareness (Table 3). Being healthcare professionals, 82% of the respondents thought that competency in knowledge regarding stem cells is very crucial, 34% of the respondents favored that MenSCs can be beneficial for the treatment of heart-related disorders, and 47% believed that MenSCs could be a potential therapeutic tool for ovarian-related diseases (Table 3).

**Table 3:** The attitude of the respondents regarding menstrual blood-derived stem cells.

Questions	Options	Number (%)
Do you feel that donation of menstrual blood is beneficial for the society?	Yes	323 (64.7)
	No	51 (10.2)
	Do not know	125 (25.1)
Do you think donation of menstrual blood for mesenchymal stem cell collection should be promoted?	Yes	364 (72.9)
	No	25 (5.0)
	Do not know	110 (22.0)
Do you think the government should do more awareness sessions regarding menstrual blood-derived mesenchymal stem cells?	Yes	407 (81.6)
	No	28 (5.6)
	Do not know	64 (12.8)
Do you think competency in stem cell knowledge is important for you as a healthcare professional?	Yes	407 (81.6)
	No	28 (5.6)
	Do not know	64 (12.8)
Do you think stem cells derived from menstrual blood can be used for the treatment of infertility?	Yes	232 (46.5)
	No	56 (11.2)
	Do not know	211 (42.3)
Do you think stem cells derived from menstrual blood can be used for the treatment of diabetes?	Yes	158 (31.7)
	No	88 (17.6)
	Do not know	253 (50.7)
Do you think stem cells derived from menstrual blood can be used for the treatment of heart diseases?	Yes	167 (33.5)
	No	91 (18.2)
	Do not know	241 (48.3)
Do you think stem cells derived from menstrual blood can be used for the treatment of ovarian-related disease?	Yes	232 (46.5)
	No	58 (11.6)
	Do not know	209 (41.9)
Do you think stem cells derived from menstrual blood can be used for the treatment of liver diseases?	Yes	178 (35.7)
	No	82 (16.4)
	Do not know	239 (47.9)
Do you think stem cell therapy is a lifesaving treatment?	Yes	249 (49.9)
	No	94 (18.8)
	Do not know	156 (31.3)

**Table 4:** Practices of the respondents regarding menstrual blood-derived stem cells.

Questions	Options	Number (%)
What is your preferred menstrual hygiene product?	Sanitary pads	442 (88.6)
	Combination: menstrual cups and sanitary pads	16 (3.2)
	Combination: tampons and sanitary pads	16 (3.2)
	Menstrual cups	6 (1.2)
	Tampons	15 (3.0)
	Tampons and menstrual cups	4 (0.8)
Are you willing to use a menstrual cup?	Yes	185 (37.1)
	No	223 (44.7)
	Do not know	79 (15.8)
	Already using it	12 (2.4)
If No, what is the reason for not using it?	Not comfortable	182 (60.2)
	Do not know the proper method to use it	47 (15.6)
	Found unhygienic	30 (9.9)
	Religious/Cultural Belief	27 (5.4)
	Unaware about it	13 (3.9)
	More expensive	4 (1.3)
Would you be willing to donate your menstrual blood?	Yes	270 (54.1)
	No	124 (24.8)
	Do not know	105 (21.0)
Have you donated your menstrual blood before?	Yes	28 (5.6)
	No	457 (91.6)
	Do not know	14 (2.8)
If yes, how frequently have you donated it?	Only once	25 (89.2)
	More than once	3 (11.0)
If not, what is the reason behind it?	Lack of knowledge in this context	234 (49.6)
	Uncomfortable with donating menstrual blood	184 (39.0)
	Lack of menstrual blood bank in my vicinity	30 (6.3)
	My religion does not allow it	23 (4.8)
Are you willing to donate your menstrual blood to a menstrual blood bank if you will receive incentive (For example: - a day off from work/studies, gift card, reimbursement from blood bank, travel fare reimbursement, etc.) in exchange for the donation?	Yes	239 (47.9)
	No	124 (24.8)
	Do not know	136 (27.3)
Will you accept therapy from menstrual blood-derived stem cell sample if you require that sort of treatment?	Yes	281 (56.3)
	No	108 (21.6)
	Do not know	110 (22.0)
Do you know any menstrual blood banks in India?	Yes	13 (2.6)
	No	326 (65.3)
	Do not know	160 (32.1)

In our study, adequate practices toward menstrual blood donation and the usage of related products were reported by 45% of the respondents (Figure 1). For 89% of the respondents, sanitary pads were the most preferred menstrual hygiene product. A total of 45% of the respondents showed unwillingness toward using menstrual cups and 60% of the respondents expressed discomfort while wearing was the primary factor for not using menstrual cups followed by a lack of knowledge regarding their proper usage (15.6%). Although 54% of the respondents showed willingness toward menstrual blood donation, the majority had not donated menstrual blood before (91.6%). Willingness toward donating menstrual blood to a menstrual blood bank was reported by 48% of the respondents provided they received incentives in the form of gift cards, reimbursements from the blood bank, and work/studies day off. Although 56% of the respondents showed acceptance toward therapy from MenSCs, only 3% of respondents had knowledge regarding menstrual blood banks in India.

Upon bivariate analysis (Table 5), the educational qualification of the respondents was found to be significantly associated (*p*, 0.002) with the respondent’s perceived attitude toward MenSCs. Respondents from the department of the medical or paramedical were found to have a significant impact (*p*, 0.033) on their knowledge about MenSCs. The occupational status of the respondents (*p* < 0.001) along with their profession (*p* < 0.001) and monthly earnings (*p*, 0.042) was also found to be significantly associated with the respondent’s attitude toward menstrual blood donation and utilization of MenSCs for therapeutic purposes.

**Table 5:** Factors associated with knowledge, attitude, and practice level of the respondents.

Factors	Sub-groups	Total *N* (%)	Knowledge score		*p* value	Attitude score		*p* value	Practice score		*p* value
			Adequate *N* (%)	Inadequate *N* (%)		Adequate *N* (%)	Inadequate *N* (%)		Adequate *N* (%)	Inadequate *N* (%)	
Age	18–24 years	334 (66.9)	294 (58.9)	40 (8.0)	0.726	177 (35.5)	157 (31.5)	0.477	148 (29.7)	186 (37.3)	0.532
	25 and above years	165 (33.1)	147 (29.5)	18 (3.6)		93 (18.6)	72 (14.4)		78 (15.6)	87 (17.4)	
Education	Undergraduate	389 (78.0)	339 (67.9)	50 (10.0)	0.107	196 (39.3)	193 (38.7)	**0.002**	175 (35.1)	214 (42.9)	0.798
	Postgraduate and PhD	110 (22.0)	110 (20.4)	8 (1.6)		74 (14.8)	36 (7.2)		51 (10.2)	59 (11.8)	
Department	Medical	313 (62.7)	284 (56.9)	29 (5.8)	**0.033**	174 (34.9)	139 (27.9)	0.389	148 (29.7)	165 (33.1)	0.246
	Paramedical	186 (37.3)	157 (31.5)	29 (5.8)		96 (19.2)	90 (18.0)		78 (15.6)	108 (21.6)	
Occupation	Employed	295 (59.1)	261 (52.3)	34 (6.8)	0.935	132 (26.5)	163 (32.7)	**<0.001**	126 (25.3)	169 (33.9)	0.164
	Unemployed	204 (40.9)	180 (36.1)	24 (4.8)		138 (27.7)	66 (13.2)		100 (20.0)	104(20.8)	
Profession	Student	287 (57.5)	258 (51.7)	29 (5.8)	0.218	131 (26.3)	156 (31.3)	**<0.001**	121 (24.2)	166 (33.3)	0.102
	Job (Government and Private)	212 (42.5)	183 (36.7)	29 (5.8)		139 (27.9)	73 (14.6)		105 (21.0)	107 (21.4)	
Monthly income	10–50 K	417 (83.6)	371 (74.3)	46 (9.2)	0.352	234 (46.9)	183 (36.7)	**0.042**	187 (37.5)	230 (467.1)	0.651
	50 K and above	82 (16.4)	70 (14.0)	12 (2.4)		36 (7.2)	46 (9.2)		39 (7.8)	43 (8.6)	

Bold values signifies that the *p*-values are significant.

## Discussion

MenSCs are pluripotent cells with the potential to replicate every 24–36 h. It is crucial to note that MenSCs exhibit characteristics associated with embryonic stem cells, giving them the apparent ability to differentiate into a variety of healthy cell types. These cells have unique characteristics that can be used as a novel therapeutic modality for the treatment of various diseases in the future including rare diseases such as amyotrophic lateral sclerosis and Alzheimer’s disease (AD) (15, 16). Hence, the purpose of this study was to assess the level of knowledge, perceived attitude, and practice of Indian healthcare professionals regarding MenSCs. To the best of our knowledge, this is the first research study in India to evaluate the awareness level and perspective of MenSCs among female healthcare professionals and to highlight the existing gaps. According to the current study conducted among Indian female healthcare workers, 49% of the respondents had knowledge regarding MenSCs. A similar study conducted among staff nurses in Tamil Nadu reported that 30% of the respondents had moderate knowledge regarding MenSCs (17), whereas a study conducted among nursing students in Government Nursing College, Jodhpur, reported a higher level of knowledge (64%) (18). A possible explanation for the variations in the level of knowledge could be the difference in the geographic region, varied sample sizes, and socio-demographic characteristics. In our study, 24% of the female healthcare workers were aware of menstrual blood banking in India. A similar study conducted among female healthcare professionals at Manipal University, Karnataka, reported that 13% of the respondents were aware of menstrual blood banks (19). On the other hand, a study conducted in Amritsar assessed both pre-test and post-test knowledge to determine the effectiveness of a teaching program among nursing students regarding menstrual blood banking and reported that during the pre-test, 93% of the respondents had just an average knowledge of menstrual blood banking, while post-test 89% had a good knowledge (20).

In total, 54% of the respondents in our study were reported to perceive a positive attitude toward the utilization of MenSCs, 65% of the respondents supported menstrual blood donation and considered it to be beneficial for society, and 73% felt it should be encouraged. Furthermore, 50% of the respondents believed that the therapeutic properties of stem cells can become a life-saving treatment modality. These findings are supported by a cross-sectional study performed in Kelantan, Malaysia, among nurses with midwifery and neonatology expertise in a tertiary teaching hospital. They found that 87% of the respondents had moderate knowledge regarding the utilization of stem cells in medicinal therapy, while 61% of the nurses reported a positive attitude toward the therapeutic potential of stem cells in medicine (21). Even though a majority of the respondents in our study were receptive toward enhancing their knowledge regarding MenSCs, some of the respondents (5.0%) were not in favor of promoting menstrual blood donation. This attitude could be due to age-old religious beliefs, socio-cultural myths, and misconceptions associated with menstrual blood that are deeply rooted in India (22).

Though the proportion of willingness regarding menstrual blood donation was found to be 54% in our study, the majority of the respondents had not donated menstrual blood before (91.6%). The major reason behind this was the lack of knowledge in the concerned field as stated by 47%. Thus, it is essential to implement multiple strategies to enhance knowledge among women regarding the donation of menstrual blood for MSCs in the form of awareness sessions, health camps, as well as interactive classes. This will not only result in a larger participation in menstrual blood donation but will also support the development of MSC therapy. This conclusion is supported by the fact that the predominant number of respondents in our study was positive toward the acceptance of therapies from MenSCs (56.3%). Similarly, a study conducted in the United Kingdom among women from local universities also showed that the majority of the women (91%) had a positive response toward acceptance of therapy derived from menstrual blood (12). The designated department of the respondents in our study (medical or paramedical) was found to have a significant association (*p* = 0.033) with knowledge regarding MenSCs. Other socio-demographic factors, such as level of education (*p* = 0.002), occupation (*p* < 0.001), and profession (*p* < 0.001), as well as monthly income (*p* = 0.042) of the respondents, were significantly associated with the attitude perceived toward MenSCs. Thus, we conclude that the occupational environment where the participants are working or studying should also be sensitized with the beneficial aspects of menstrual blood and the advantages of using its related products. This can be a potential strategy to address the misconceptions related to menstruation in the Indian scenario thereby limiting the stigma associated with it.

As the most preferred menstrual hygiene product was sanitary pads, which was reported by 89% of the respondents, 45% of the respondents showed unwillingness toward the usage of menstrual cups. Their predominant reason was discomfort, as reported by 60% of the respondents. However, because a significant proportion of female healthcare workers were willing toward menstrual blood donation, they should be encouraged and educated regarding the application and the superadded benefits of a menstrual cup. A preliminary study reported that women who were learning the menstrual cup application for the first time showed better experience outcomes with guidance and positive support (23). Therefore, providing a menstrual cup with assistance during the initial use might result in a more favorable first-hand experience for Indian women who have never used a menstrual cup before and may increase their likelihood of using the product on a regular basis. MenSC donation should be promoted as it can benefit society as a whole as well as make individual women feel positive toward this normal physiological process.

### Limitations

This study was conducted specifically among female healthcare professionals, which are limiting factors. Focusing on one particular profession or community will not be enough to determine the gravity of the scenario. Another limitation of this study was the social desirability bias that can be introduced due to the self-constructed nature of the questionnaire. To avoid such a bias, validated subscales should be developed to assess the knowledge, attitude, and practice of the general populace regarding important public health issues prevalent in the LMICs such as menstrual hygiene and sanitation. Furthermore, due to the cross-sectional design, this study cannot provide the longitudinal impact on the attitude and practice affecting the knowledge of the participants regarding MenSCs as it lacks follow-up surveys. Therefore, more extensive studies should be conducted at all socio-demographic levels covering all genders from all economical strata, which will depict the actual picture regarding the awareness and the perceived attitude toward menstrual blood donation and MenSCs.

## Conclusion

This study highlights that there is only limited knowledge regarding MenSCs among women healthcare workers with a lack of adequate practice for the same. In order to encourage women to menstrual blood donation, healthcare workers must know how to foster a good attitude toward menstruation both among themselves and within the community. As a future practice, the key focus will be to instigate female healthcare workers with adequate knowledge and awareness about MenSCs because they are the key representatives in promoting new life-saving modalities and treatments in the community. The responses of the female healthcare workers in our study toward MenSCs should be taken as a positive factor to initiate further studies that are required in the general population to assess and improve the knowledge and attitude toward MenSCs. This will eventually encourage researchers to work on MSCs, their storage, and therapeutic use. The enhancement of knowledge and awareness regarding the potential benefits of MenSCs would not only help in dissipating the age-old myths associated with menstruation but will also benefit society as a whole.

### Implications

The results of this study showed that there is a need to create awareness regarding MenSCs therapy because a majority of the women showed willingness toward menstrual blood donation. The healthcare personnel should be equipped in conducting various health education programs and camps at the community level for reinforcement of knowledge, positive attitude, and positive practice associated with menstruation and its related products.

## Data availability statement

The original contributions presented in the study are included in the article/supplementary material, further inquiries can be directed to the corresponding author.

## Ethics statement

The studies involving human participants were reviewed and approved by SRM Medical and Research Ethics Clearance Subcommittee. The patients/participants provided their written informed consent to participate in this study.

## Author contributions

KK: conceptualization. DN: data analysis. LM: draft writing. SS: data collection. MS: visualization. ST: validation. RJ: supervisor. KR: writing, reviewing and editing. All authors contributed to the article and approved the submitted version.

## Conflict of interest

The authors declare that the research was conducted in the absence of any commercial or financial relationships that could be construed as a potential conflict of interest.

## Publisher’s note

All claims expressed in this article are solely those of the authors and do not necessarily represent those of their affiliated organizations, or those of the publisher, the editors and the reviewers. Any product that may be evaluated in this article, or claim that may be made by its manufacturer, is not guaranteed or endorsed by the publisher.
